# Round the Hospitals

**Published:** 1919-03-22

**Authors:** 


### ROUND THE HOSPITALS.

The King has been pleased to appoint to the Order of the British Empire Miss Louisa Mary Stewart, R.R.C., matron Q.A.I.M.N.S., Dover Military Hospital, C.B.E. (Military Division). The King has been pleased to award the Royal Red Cross to the following ladies of the Nursing Services for valuable services in connection with the war:—Bar to the Royal Red Cross: Miss A. S. Bond, R.R.C., matron Barnet War Hospital; Miss A. F. Byers, R.R.C., matron Devonport Military Hospital; Miss G. M. Chadwick, R.R.C., matron Hounslow Military Hospital; Miss J. Clay, R.R.C., acting matron County of Middlesex War Hospital, Napsbury; Miss R. Cox-Davies, R.R.C., principal matron T.F.N.S., No. 1 London General Hospital; Miss M. E. Davies, R.R.C., matron King George Hospital, Stamford Street; Miss M. C. S. Knox, R.R.C., matron Curragh Camp Military Hospital;

**ROUND THE HOSPITALS—(*continued*).**

Miss H. W. Reid, R.R.C., matron Royal Victoria Hospital, Netley; Miss L. E. C. Steen, R.R.C., matron Reading War Hospital; Miss L. W. Tullon, R.R.C., matron Guildford War Hospital. Royal Red Cross, First Class.—Miss S. E. Bland, matron Poplar Hospital for Accidents; Miss L. Ellis, matron Military Families' Hospital, Portsmouth; Miss C. E. A. Harries, acting matron Military Hospital, Ripon; Miss F. E. Manfield, A.R.R.C., acting matron Dartford War Hospital; Miss E. E. O'Connell, acting matron Croydon War Hospital; Miss E. Schafer, A.R.R.C., acting matron Military Hospital, Weymouth; Miss E. Thacker, A.R.R.C., sister Military Hospital, Dover; Miss M. L. Tyndall, matron Military Families' Hospital, Devonport; Miss B. Walker, matron Prees Heath Military Hospital, Whitchurch. Royal Red Cross, Second Class.—Miss L. Fletcher, sister King George Hospital; Miss Maude Plant, staff nurse King George Hospital; Miss G. C. Quentrall, sister 5th London General Hospital; Miss L. M. Wass sister Queen Alexandra Military Hospital.

At the Investiture held in the Ball-room at Buckingham Palace on March 15, the King personally conferred the Royal Red Cross, First Class, on Matron Alice Nye, late Q.A.I.M.N.S.R., India; and on Matron Inga Johnson and Sister Helen Shearer, Canadian A.N.S. His Majesty conferred the Royal Red Cross, Second Class, on Sister Ethel Hutchings, Civil Nursing Service, India; on Sister Ethel Giddings, Sister Annie West, and Staff Nurse Alma Furniss, Australian A.N.S.; and the Military Medal on Sister Janet Williamson, Canadian A.N.S. Mrs. Margaret Sheild and Miss Annie Wavell, V.A.D., received the honour of the Royal Red Cross, Second Class.

The following protest signed "Members of Q.A.I.M.N.S.R.," which appeared in Wednesday's *Times* claims the immediate attention of the authorities: "The members of Queen Alexandra's Imperial Military Nursing Service Reserve would like to bring before the public the unfair way in which they are being demobilised. They are being given forty-eight hours' notice, and after that pay and allowances cease. Many of us have served now for four years, and never having had proper holidays, are unfit to start work again immediately. We are a body of women working for our living, and are not in a position to be dismissed at so short notice. Many have no parents or homes, and will have to go into lodgings and pay for them. Are we not entitled to some consideration in the form of one month's wages and allowances in lieu of a month's notice? The War Office expect one month's notice from any one leaving the Service, and the extra £20 added to our salaries in September 1916 had to be refunded." What have the authorities to say?

The Liverpool Centre of the College of Nursing is pressing for better representation on the Council of the College. At present there are only five provincial members, and it is suggested that a definite number of representatives might be allotted to the provinces, which should be divided into districts accordingly, "the members in each district to be free to vote for any candidate *in that district* without coercion." The resolution passed at a large meeting with over 100 members present was forwarded to headquarters for inclusion in the agenda of a Council meeting.

We wish the register of the College of Nursing all success, but we fear it will be a costly business to get it printed, and should its publication be delayed we could with equanimity await its production till things are easier in the trade. At the present period of rapid growth in the number of registered nurses any printed register must be out of date in the course of a few weeks after publication.

Some significant figures were quoted in Parliament on March 10 by Mr. Leonard Lyle, who asked the Secretary of War whether 500 doctors and 2,000 nurses might not at once be released from the Army on indefinite leave for service among the civilian population. Mr. Lyle stated that there were still nearly 10,000 doctors and over 20,000 nurses in the Army, and that if civilians were provided with nurses in the same proportion as the military, approximately 270,000 nurses would be required at home, whereas there were approximately only some 40,000 or 50,000 trained nurses altogether. Captain Guest, in reply, stated that since the armistice 7,441 trained and untrained nurses had been demobilised, but declined to consider the proposal to release doctors and nurses pending demobilisation.

The discussion on "Uniform, its Uses and Abuses," which has taken place in connection with the Cambridge College of Nursing Centre at Addenbrooke's Hospital, opened up many interesting points in this vexed question. The present style of cloak and bonnet out-door uniform was voted indefensible, and something on the lines of the much-admired uniform worn by members of the Canadian and Australian Military Nursing Services was suggested, though with the proviso that a long coat must be allowed to district nurses. Two conclusions were reached, the first, that institutional nurses ought not to be permitted to wear their ward dresses out of doors but should be provided by the authorities with an optional uniform; the second that nurses in training ought not to wear out-door uniform at all, since at the outset of their training they do not always realise the dignity of their profession in public places, nor are they able to render efficient help when appealed to in emergencies. In our judgment the meeting put the finger on the main cause of the "nurse tales" so greedily caught up and repeated by the public. To label young women from the first moment of their arrival in hospital as trained nurses in the eyes of the public by equipping them in nurse's uniform is to open the door to the

**ROUND THE HOSPITALS—(*continued*).**

gravest abuse. A plain serge coat and skirt with simple hat would be far more suitable for probationers than their present garb.

THE Army Council Instruction A.C. 1, 65/19, on which we have commented, is rousing determined protest in many quarters. An extraordinary general meeting of Incorporated Society of Trained Masseuses members has been held in Dublin to discuss the situation, and representatives were present from the Special Military Surgical Hospital, Blackrock, King George V. Hospital, and the Castle Red Cross Hospital. The chair was taken by Miss Shuter, and Miss Despard explained to a deeply interested audience the steps which are being taken to get the unfortunate Order for the admission of unqualified masseuses to the Army Service revoked and raise the status and pay of the qualified masseuse.

AT the Maidstone Poor Law Infirmary an antiquated system is still in force, under which each nurse spends her own mess allowance and has her individual meals cooked either by an inmate or by herself in the infirmary. This wasteful plan has been reported against by the Local Government Board inspector lately, and a recommendation made that allowances for the nurses' diet should be pooled and put under the control of the matron or senior sister. The inspector, Mrs. Andrews, reports also that an additional certificated sister is needed to obviate the necessity for the matron to relieve the sister on duty by day. It would seem that the Maidstone Guardians need some new people, especially women, on their Infirmary Committee. It is not creditable to this body that such an unsatisfactory system of messing should have continued up to the present.

IT seems probable that other Red Cross organisations will be eager to follow the good example set by the Camberwell Division in getting affiliated to King's College Hospital, and will offer their services to institutions for the sick within their immediate neighbourhood. It will be deplorable if, with all this goodwill ready to hand, the Poor-Law infirmaries fail to attract any of it to their assistance. The chronic and children's wards in many Poor-Law establishments sorely need just those extra pairs of hands which a good Red Cross detachment ought to be able to supply. Help in washing and feeding chronic patients and in the care of infants and young children is quite within the compass of voluntary and part-time workers. They would introduce a fresh current of interest into these wards, where probationers tell us they become hard and indifferent early in their career because they are always in a hurry. There are many Poor-Law infirmaries in which the matron succeeds miraculously in attaining a standard equal to that of a good voluntary hospital. But even in these the strain of understaffing is severely felt. Lack of accommodation prevents these institutions increasing the staff, and the introduction of the V.A.D. would in many instances solve a pressing problem.

THE twenty-eighth annual report of the Nurses' Co-operation records a hard but eminently successful year's work. The fee for all ordinary cases stands now at £3 3s. a week, with a guinea a week extra where massage is required, and an extra half-guinea weekly in sundry other contingencies, besides laundry expenses and all allowances. The nurses receive the whole of their earnings, subject only to a deduction of 5 per cent. (1s. in the £) in the case of those who joined before the end of 1908, and 7½ per cent. for the others. The general staff is made up of 450 fully trained nurses, thirty-five asylum trained for mental patients, and twenty-three nurses, eligible for election, working on probation for six months. The gross earnings of the staff last year far exceeded those in any previous year; they reached a total of £57,284, of which £53,637 was paid to the nurses. Three sisters died during the year—Sister Gledhill (in Palestine), Sister J. Parsons, and Sister G. Stephenson (invalided home from Salonika). Thirteen of the staff married during the year. Several, among whom are Sister G. M. Porter, Sister M. Paul, and Sister S. Baxter, have been awarded the Royal Red Cross. Many are still abroad, and their return is eagerly awaited by the Secretary, who, needless to say, is overwhelmed with applications for nurses at the moment. Few movements for the benefit of nurses have had more effect in securing justice for the profession than co-operation. It has obtained widespread imitation, and we look forward to the time when the principles underlying the Nurses' Co-operation, well and wisely laid down at its inception, will be recognised and acted on in every nurses' institute in the kingdom.

WE learn with the greatest pleasure from the secretary of the Ancoats Hospital, Manchester, that at the meeting of the Committee of Management held on February 26 the matron asked for permission to arrange that the nurses of this hospital should have one full day off in seven. The request was granted unanimously by the Committee, and Ancoats Hospital from now is associated with hospitals in the van of progress. The hospital contains 130 beds. The matron is Miss Maud Earl, and the training-school, which bears a high repute, consists of twenty-six nurses and probationers under ten sisters. This number will now, of course, be increased, and since Manchester is notoriously very short of trained nurses, the enlargement of the training-school may be considered in itself a public benefit.

THE meeting at the Mansion House, at which the Lord Mayor presided, on March 13, to promote the raising of a fund for founding a club for the members of the Royal British Nurses' Association, definitely disposes of the absurd theory that it is ignominious for public moneys to be contributed for the benefit of nurses. Only a very few persons ever dreamed of objecting to the offerings spontaneously given to the Nation's Fund for Nurses, and these have now evidently come to realise the magnitude of their folly. Some excellent speeches were made

**ROUND THE HOSPITALS—(*continued*).**

at the meeting, and, as the Lord Mayor said, there is plenty of room for another Nurses' Club in London.

Among the beds at the Elizabeth Garrett Anderson Hospital for which appeal for endowment was made last year, was one to be called the Nurses' and Masseuses' Bed. We are informed that the endowment is now complete, and grateful thanks are offered to all who have collected for or contributed towards the bed. It proved a popular fund from the first, and has met with widespread support from nurses and their patients.

The promoters of the Pro Patria Day Nursery at 121-123 Bishop's Road, Bethnal Green, are appealing for £1,000 in order to complete an establishment greatly needed in the midst of a slum neighbourhood and admirably managed on modern lines. The nursery accommodates seventy babies and children under school age, and has a little convalescent home in Sussex for the more ailing of its clientele. The mothers pay 9d. a day, and each child has three good meals for that.

An unusual celebration took place at the Royal Victoria Infirmary, Newcastle, on March 13, when Sister Whitfield, after thirty-three, and Sister Nichol, after thirty-two years' service, received the thanks of their colleagues and superior officers, with handsome presents in recognition of their loyalty and devotion to the interests of the institution. Lt.-Col. Richardson, who presented a gold watch to Sister Whitfield, recalled the fact that he was her first house surgeon and spoke gratefully of the loyal services she ever rendered, which had brought her to believe that "her honoraries, house surgeons, and dressers were the best in the hospital." Dr. Hume, who presented Sister Nichol with a solid silver teapot and tea spoons, paid a similar tribute to her high qualities as a ward sister. Illuminated books containing the names of all the subscribers were handed to both ladies.

Miss Nutt has resigned her position as sister at the West Bromwich Poor-Law Infirmary on account of ill-health, and in recognition of her long services the Guardians have applied to the Local Government Board for permission to add six years to her term and thus raise her retiring pension to £60 instead of £51 per annum. We are glad to see that the Guardians are also applying for leave to raise the sisters' salaries to £40 on appointment, £43 in the second year, and £45 subsequently.

The Cathedral nurses, established in Newcastle thirty years ago by Bishop Lloyd, have been overstrained during the influenza epidemic, and what is even worse, have been compelled to leave many of the poor unvisited. The circumstances have brought to notice the disgraceful housing conditions of the city. In one house where phthisis claimed its victim, green ooze stood out upon the walls. In another, the mother with pneumonia and a girl with phthisis were in one bed, and six children lying top to toe in another bed with no one to do anything for them. The lady superintendent, Miss Abraham, has offered to train voluntary workers to be of use in this emergency by giving them instruction in the difficulties to be surmounted, ventilation, disinfection, etc. It seems to us that what is most needed in Newcastle is not more nurses but new members of the City Corporation, pledged to sweep away the slums and open up a prospect of decent living for workers. Nurses were never intended to act as a sop to public sentiment, and save the ratepayers from drastic improvements long overdue.

The value of the sister-tutor in supervising the education of probationers and making their training a real preparation for future duties is already established wherever it has been tried. But the training-schools which can afford to pay an adequate salary for a sister whose primary functions are educational do not include the great majority. It is now a recognised "plank" in the policy of the College of Nursing to facilitate the great educative reforms which can only be carried out under the guidance of a tutor devoting her entire energies to the probationer. In the advertisement of the Nation's Fund for Nurses inserted in the leading papers the purposes of the Fund are defined, and it is stated that one of its leading aims is "to equip a specially trained sister-tutor for every training-school for nurses in the kingdom." Many training-schools which are ready to introduce the tutorial system are hampered by lack of women trained for this particular work, and already there is much to encourage women of higher education to enter the nursing profession with a view to taking up such appointments.

A deputation from the Women's Sanitary Inspectors' and Health Visitors' Association was received by Mr. Willis, Assistant Secretary to the Local Government Board, on March 14, on behalf of Dr. Addison, and brought to notice various questions relating to the salaries, war bonuses, and holidays of the workers they represented, as well as the need for a single statutory qualification for health visitors. A sympathetic reply was given to the views expressed by the deputation, of whom Miss Orange, chairman of the Association, was spokeswoman.

**☛ It is our invariable practice to pay for all items of news which we may publish. The minimum payment is 5s. Paragraphs of about twenty lines in length—that is to say, of 150 words or less—are preferred. Contributions should be addressed to The Editor, The Hospital, 28 and 29 Southampton Street, Strand, London, W.C. 2, and, to be in time for the current week's issue, should reach him by the first post on Mondays.**

